# Early detection of subclinical heart disease via nonlinear heart rate variability in a doxorubicin-induced cardiomyopathy experimental model in dogs

**DOI:** 10.3389/fvets.2025.1725439

**Published:** 2026-01-13

**Authors:** Mizuki Hasegawa, Mayuko Sasaki, Yui Umemoto, Rio Hayashi, Akari Hatanaka, Marino Hosoki, Ahmed Farag, Lina Hamabe, Kazumi Shimada, Katsuhiro Matsuura, Tomohiko Yoshida, Ken Takahashi, Ryou Tanaka

**Affiliations:** 1 Department of Animal Medical Center, Tokyo University of Agriculture and Technology, Tokyo, Japan; 2 Department of Veterinary Surgery, Tokyo University of Agriculture and Technology, Tokyo, Japan; 3 Department of Veterinary Clinical Oncology, Tokyo University of Agriculture and Technology, Tokyo, Japan; 4 Department of Small Animal Clinical Sciences, College of Veterinary Medicine, University of Florida, Florida, FL, United States; 5 Department of Small Animal Medical Center, Obihiro University of Agriculture and Veterinary Medicine, Obihiro, Hokkaido, Japan; 6 Department of Pediatrics, Juntendo University, Urayasu Hospital, Chiba, Japan

**Keywords:** cardiology, dogs, Holter electrocardiography, parasympathetic nerves, Poincaré plot, sympathetic nerves, two-dimensional speckle-tracking echocardiography

## Abstract

**Background:**

Echocardiography is the first choice for assessing the structure and function of the heart, but it is unclear for detecting subclinical changes. In recent years, abnormal heart rate variability (HRV) has received attention for its ability to identify patients at risk for developing heart failure. HRV analysis in veterinary medicine is predominantly limited to linear analysis, which primarily reflects advanced heart disease. In contrast, nonlinear HRV analysis holds the potential for early detection of heart disease, but its quantitative evaluation remains rare.

**Objectives:**

This study aimed to evaluate the feasibility of using HRV for the early heart disease detection in clinical settings, with a focus on doxorubicin (DXR)-induced myocardial damage in dogs.

**Animals and methods:**

Six healthy female dogs with no abnormalities on physical examination, blood pressure, electrocardiography (ECG) and echocardiography were selected in this study. The dogs had an average age of 1.2 years and an average body weight of 8.1 kg. After recording blood pressure, ECG and echocardiography, the dogs were fitted with a Holter ECG, and measurements were taken for 2 days. Following the removal of the Holter ECG, DXR at 30 mg/m^2^ was administered over 30 min, repeated every 3 weeks, up to a maximum cumulative dose of 180 mg/m^2^. Each measurement was taken before the first and after the final DXR dose.

**Results:**

There were no changes in recommended parameters of left ventricular systolic function (FS: 34.4% [33.9–42.8] vs. 37.8% [34.7–42.8], *p* = 0.73, GLS EN: −19.1% [−21.3 – −17.5] vs. −18.0% [−19.3 – −17.3], *p* = 0.68). However, the Poincaré plot of nonlinear HRV significantly reflected increased sympathetic activity (SD1/SD2: 0.58% [0.57–0.60] vs. 0.42% [0.40–0.45], *p* = 0.008, SD2/SD1: 1.8% [1.76–1.82] vs. 2.5% [2.3–2.7], *p* = 0.008).

**Conclusion:**

The finding that nonlinear HRV analysis reflected early increased sympathetic activity associated with DXR administration in dogs is an important step forward in enhancing the clinical application potential of HRV.

## 1 Introduction

The significance of early detection of heart disease lies in improving outcomes through timely treatment interventions. Currently, echocardiography is the preferred method for assessing the structure and function of the heart. However, echocardiography is limited in detecting structural or functional changes in the heart at the subclinical stage of disease, making it challenging for veterinarians to diagnosis or determine treatment during the early stage (1–3).

To address the difficulty in evaluating the subclinical stage of heart diseases, tissue Doppler imaging (TDI) and two-dimensional speckle-tracking echocardiography (2D-STE) have been proposed as methods capable of detecting issues at the early stage of the disease. However, TDI has the disadvantage of being unable to assess diastolic function when an arrhythmia is present or if the heart rate (HR) is too high (4, 5). Fractional shortening (FS) is a commonly used parameter for the early detection of left ventricular systolic dysfunction in dogs via conventional echocardiography. However, it only assesses myocardial shortening in specific segments and is also influenced by the loading conditions (6). These disadvantages are overcome with 2D-STE. However, 2D-STE requires high-quality images with a high frame rate, and in addition to these technical challenges, it involves offline analysis (6, 7). Therefore, 2D-STE is not suitable for routine clinical use because it is more time-consuming than the conventional echocardiography. As a result, current echocardiographic methods face challenges in predicting and early detecting heart disease. Based on the above, there is a need to establish an examination method that can predict and detect heart disease at an early stage as an alternative to echocardiography, even in veterinary medicine.

In recent years, abnormal heart rate variability (HRV), specifically an increase in sympathetic activity within HRV parameters, has gained attention for its ability to identify patients at risk of developing heart failure (8). The fact that HRV can be measured in the comfort and safety of the home, where pets feel most comfortable, makes it an ideal method to address various challenges faced in veterinary clinical setting. In addition to its relevance in cardiology, autonomic indices have also been applied to evaluate nociception and comfort in animals. Previous studies have demonstrated their potential as physiological markers for several applications, including the assessment of the analgesia–nociception balance under anesthesia and postoperative pain management (9, 10). Therefore, we decided to focus on HRV as we believe it could be applied in veterinary medicine.

Research on HRV in veterinary medicine began with studies in healthy dogs, using pharmacological blockade of sympathetic and parasympathetic nervous activity to determine the origin of frequency analysis peaks (11). Later, HRV analysis was developed to evaluate heart disease, and a study using linear HRV analysis to predict sudden death in Doberman Pinschers with dilated cardiomyopathy (DCM) suggested that parasympathetic nervous system indicators in time-domain and frequency-domain analyses decreased only in the severe DCM group (12). Similarly, a study in Boxer dogs, which are predisposed to arrhythmogenic right ventricular cardiomyopathy, confirmed a decrease in parasympathetic indicators in time-domain analysis only in groups with arrhythmogenic right ventricular cardiomyopathy accompanied by congestive heart failure (13). In addition, dogs with myxomatous mitral valve degeneration (MMVD) were divided into two groups based on the presence or absence of congestive heart failure. The results of a time-domain analysis showed that only the MMVD group with congestive heart failure exhibited a decrease in parasympathetic nerve activity (14). These reports have highlighted the difficulty of detecting early changes in heart disease using conventional linear analysis.

In previous human studies of subjects without heart failure, nonlinear analysis was identified as the most effective indicator for predicting incident heart failure during the observation period. As a result, there has been increasing interest in nonlinear analysis in recent years (8, 15). Against this background, nonlinear analysis has gained attention in veterinary medicine, and reports using nonlinear analysis are gradually increasing. Studies applying nonlinear analysis in veterinary have shown that the parasympathetic index is elevated at rest and that measurements during activity influence geometric analysis. Additionally, there are reports that classify dogs with MMVD by stage and evaluate the shape of the Poincaré plot (16, 17). However, quantitative assessment of heart disease using nonlinear analysis is still rare, and the use of HRV analysis for heart disease in veterinary medicine is currently limited to linear analysis, making it difficult to use for early detection. Based on this background, the aim of this study was to evaluate the feasibility of using HRV for early detection of heart disease in clinical settings. To achieve this, we focused on doxorubicin (DXR) - induced myocardial damage, for which no effective consensus has been reached, despite the recognition of the importance of early detection.

Cardiotoxicity is a common complication of chemotherapy. Although DXR is known to cause cumulative cardiotoxicity, it is used in both human and veterinary medicine for the treatment of various malignancies due to its efficacy and broad indications (18). In humans, the cumulative dose of DXR is usually limited to 400–450 mg/m^2^, although it is known that some myocardial damage can occur at doses well below this maximum tolerated dose (19). In dogs, 18.3% showed myocardial damage at a cumulative DXR dose of 210 mg/m^2^ and several dogs developed damage at 90 mg/m^2^ or less (20). Dogs are more sensitive to DXR cardiotoxicity than humans, with greater individual variation and more cautious dosing. However, there is no consensus on the best way to monitor DXR-induced myocardial damage. In humans, guidelines recommend echocardiographic assessment of left ventricular systolic function using ejection fraction (EF) to identify patients at risk of developing myocardial damage (21). However, changes in EF often lag in the early detection of cardiotoxicity (22). Therefore, methods to detect early myocardial damage by measuring serum cardiac troponin have been explored (23). Cardiac biomarkers indicate an increased risk of myocardial damage, but there are no data to distinguish whether the damage is reversible or progressive (21).

Early detection of DXR-induced myocardial damage suggests that the rapid initiation of treatment can restore cardiac function (24). The objective of this study was to evaluate the feasibility of using HRV for the early detection of heart disease in clinical settings, with a focus on DXR-induced myocardial damage in dogs. We aimed to investigate whether nonlinear HRV, which is related to the essence of life, could reflect myocardial damage earlier than conventional echocardiography or linear HRV. We hypothesized that HRV could detect signs of DXR-induced myocardial damage before it occurs and that changes in HRV index would follow.

## 2 Materials and methods

### 2.1 Animals

For this intervention study, dogs were selected from experimental Beagle dogs at Kitayama Labes in Nagano, Japan. Healthy female dogs with no abnormalities on physical examination, blood 1 year of age were chosen, as comparing healthy dogs under 1 year of age, during the growth process, with healthy dogs over 1 year of age, who have stable growth, suggests that heart rate (HR) is higher in dogs under 1 year of age (25). In addition, dogs with a body condition score (BCS) of 5, as provided by the World Small Animal Veterinary Association, were selected because obesity affects HRV (26). G*Power (The G*Power Team, G*Power 3.1.9.7 version, Germany) was used to calculate the sample size needed for the study. We set the parameters as following; *α* = 0.05, 1-*β* = 0.8, and effect size (d-family) = 0.5. To comply with the Animal Research: Reporting of *In Vivo* Experiments (ARRIVE) guidelines, particular attention was paid to the ethical justification of inducing myocardial damage. Because the objectives of this study could not be achieved using non-animal alternatives, the use of experimental animals was considered essential. All procedures were designed to minimize pain, distress, and burden to the animals. Moreover, since doxorubicin administration has the potential to cause sustained discomfort, a humane endpoint was predefined so that any dog showing signs of undue suffering would be euthanized even before reaching the maximum cumulative dose. From this ethical perspective, inducing myocardial damage in a larger number of dogs was not acceptable. Although G*power analysis indicated that at least 34 animals would be required, we reduced the sample size to six dogs—the minimum number needed for statistical analysis—in accordance with the 3Rs principle, particularly Reduction. In addition, scientific validity was ensured while minimizing animal use by adopting a within-subject design in which each dog’s baseline value served as its own control. All experimental dogs were handled in accordance with the guidelines established by the Institutional Animal Care and Use Committee of TUAT (Approval number: R05-141).

### 2.2 Experimental design

This study was conducted as a prospective interventional study using convenience sampling. The study variables included blood pressure, ECG, echocardiography and HRV parameters. After measuring blood pressure, ECG, and echocardiography, the dogs were fitted with a Holter ECG, and measurements were taken for 2 days. After removal of the Holter ECG, DXR (30 mg/m^2^) was administered over 30 min. This cycle was repeated at 3-week intervals until a maximum cumulative dose of 180 mg/m^2^was reached. Measurements were performed at the beginning of DXR administration (Pre) and after the last DXR administration (Post).

### 2.3 Doxorubicin

The cumulative dose of DXR was set at 180 mg/m^2^, the upper limit of clinical use in dogs (18). A 22G x 1″ indwelling needle (B. BRAUN, Melsungen, Germany) was placed in cephalic vein. DXR (Nippon Kayaku Co. Ltd., Tokyo, Japan) 30 mg/m^2^ was diluted with saline and administered over 30 min. This was repeated every 3 weeks for a total of six administrations.

### 2.4 Blood pressure test

The sphygmomanometer, manufactured by FUKUDA M-E KOGYO CO., LTD. (BP100D, Tokyo, Japan) was used. The measurement was performed using oscillometry. The dog was placed in right lateral recumbency, the cuff (No. 3) was wrapped around the base of the tail, and the dog was examined at rest in a dark room. The measurement was performed six times, and the average systolic blood pressure value was used.

### 2.5 Electrocardiography

The electrocardiograph, manufactured by FUKUDA M-E KOGYO CO., LTD. (CARDISUNY D700, Tokyo, Japan) was used. The dog was placed in right lateral recumbency and examined at rest. Electrodes were placed at the elbow for the forelimb and at the knee for the hindlimb in the following order: right front, left front, right hind, left hind. Measurements were taken after 3 min of observation, and the waveform was recorded when it became stable.

### 2.6 Echocardiography

A Prosound F75 (FUJIFILM, Tokyo, Japan) equipped with a 5 MHz sector probe was used. The dog was placed in the lateral position and examined at rest.

#### 2.6.1 Conventional echocardiography

FS was calculated from the right parasternal short-axis view at the chordae tendineae level using M-mode. Mitral annulus systolic velocity (s’) by TDI was recorded from the left parasternal four-chamber view. TDI measurements were performed at two locations by placing a sample volume on the septal and lateral wall sides of the mitral annulus at each location. Five heartbeats were acquired for the measurement, the highest and lowest values were excluded, and three heartbeats were used for the analysis.

#### 2.6.2 Two-dimensional speckle-tracking echocardiography

The frame rate was set to 80 FPS or higher and 6 heartbeats were recorded. DAS RS-1 (FUJIFILM, Tokyo, Japan) was used for analysis. Global circumferential strain (GCS) was analyzed using the right parasternal left ventricular short axis views at the levels of the tendon cord (Basal), papillary muscle (Mid), and apical (Apical) levels. GCS was assessed in the endocardium (EN), epicardium (EP), and the mid-layer between endocardium and epicardium (MID). Global longitudinal strain (GLS) was assessed for the EN using the left parasternal four- chamber view.

### 2.7 Holter monitoring

The Holter electrocardiograph used in this study was manufactured by NIHON KOHDEN CORPORATION (RAC-5203, Japan). Prior to electrode placement, the dogs’ thoraxes were shaved vertically from the sternal scape to the xiphoid process and horizontally around the fifth and sixth intercostal spaces, then cleaned with alcohol. Disposable ECG electrodes (XUNDA BRAND, China) were positioned using the M-X induction method and the R-L induction method, which is perpendicular to the M-X method. Subsequently, the induction cords were attached to the electrodes, CH1- was placed on the manubrium of the sternum, CH1 + on the xiphoid process, CH2- on the right 5th ~ 6th intercostal space, CH2 + on the left 5th ~ 6th intercostal space, and a ground electrode in the middle (27). Both M-X and R-L leads were recorded. To secure the Holter recorder and leads to the dog, an elastic bandage and a vest were utilized (27). Holter electrocardiogram recordings were conducted by veterinarians and clinical laboratory technicians in the TUAT laboratory for 48 h, during which the animals were allowed free movement within the enclosure.

### 2.8 Heart rate variability

For the 48-h Holter ECG measurements, the period from 12 a.m. on the first night to 8 a.m. on the second day was earmarked for analysis as sleep HRV. This period was based on the results of our last study describing the usefulness of HRV during sleep (27). HRV analysis was conducted using the Juntendo University algorithm with MATLAB (MathWorks, R2022a, United States). Both traditional linear analysis and a newer nonlinear analysis method were employed under the following conditions.

#### 2.8.1 Linear heart rate variability

HRV variables for frequency analysis include total power (TP, 0–0.4 Hz), ultra-low frequency (ULF, 0–0.00333 Hz), very low frequency (VLF, 0.00333–0.04 Hz), LF (0.04–0.15 Hz), and HF (0.15–0.4 Hz). The parameters utilized in this study were normalized high frequency (nHF) and LF/HF ratio. Normalization eliminates much of the significant within-subject and between-subject variation, resulting in increased reproducibility (28). Therefore, HF and LF were normalized using the equations nHF = HF/(LF + HF) and normalized LF = LF/(LF + HF) (28). Regarding time-domain analysis in linear analysis, SDNN and RMSSD were employed. The standard deviation of the average NN intervals (SDANN), SDNN index and pNN50 were not included in the analysis for the following reasons (29). For SDANN, it is correlated with SDNN and is generally considered redundant. The SDNN index only estimates variability due to factors affecting HRV within a five-minute period. For pNN50, RMSSD typically provides a better assessment of respiratory sinus arrhythmia (RSA) and it is often preferred over pNN50.

#### 2.8.2 Nonlinear heart rate variability

**Figure 1:**
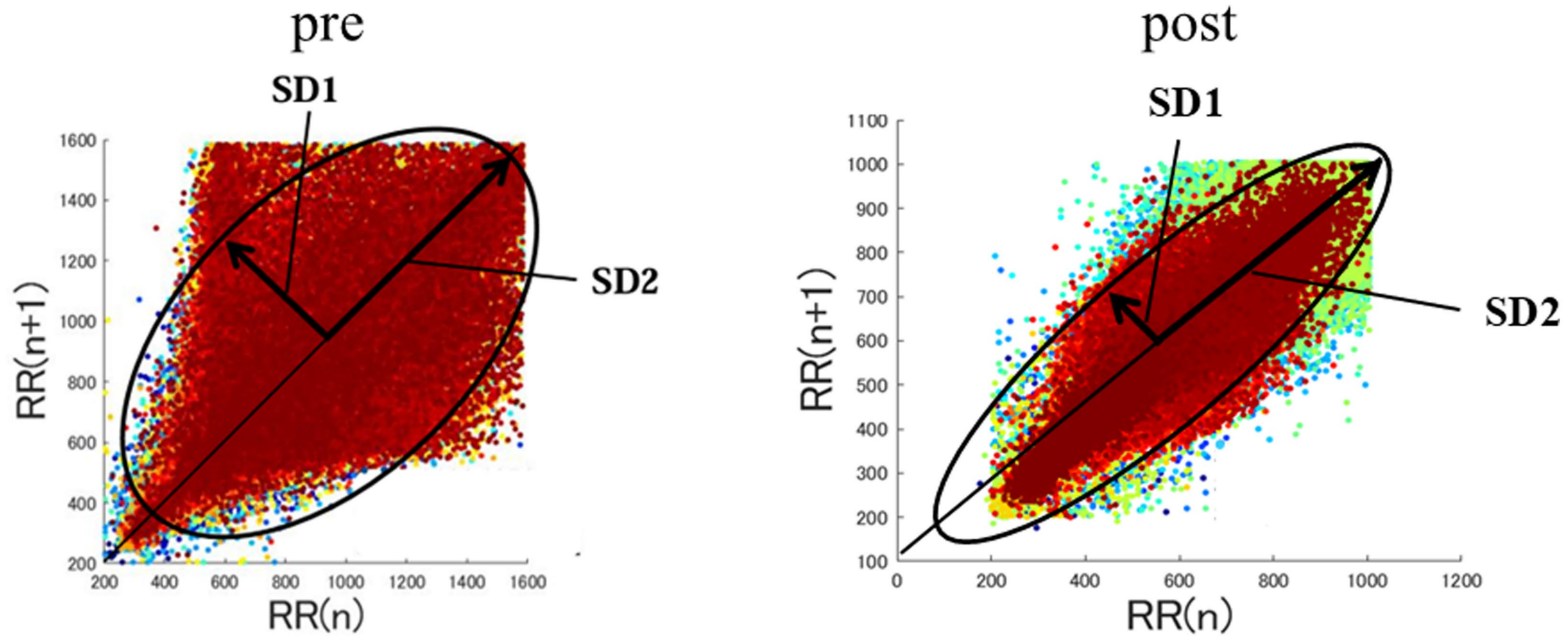
Poincaré plot. This plot shows the HRV pattern obtained from the same experimental animal before and after doxorubicin administration. In the geometric method of nonlinear analysis, indices such as SD1 and SD2 can be visually evaluated using a Poincaré plot. SD1 shows how much the points are scattered in the direction of the minor axis of the ellipse. SD2 shows how much the points are scattered along the long axis of the ellipse. Greater variability between adjacent RR intervals, reflecting stronger parasympathetic activity, results in a wider SD1 spread. In the present study, we observed low SD1/SD2 values and high SD2/SD1 values, indicating enhanced sympathetic activity following doxorubicin administration.

For the nonlinear analysis, both geometric and phase- rectified signal averaging (PRSA) were employed. Geometric analysis involved plotting a Poincaré plot by graphing every R-R interval against its preceding interval, thus creating a scatter plot (Figure 1). This plot can be analyzed by fitting an ellipse to the plotted points. The standard deviation of the distance of each point from the y = x axis was measured as SD1 (width of ellipse), while the standard deviation of the distance of each point from y = x + mean R-R interval was measured as SD2 (length of the ellipse) (16, 29–33). The ratio SD1/SD2 and SD2/SD1 were measured to assess autonomic balance. A signal processing technique called PRSA was used to quantify acceleration capacity (AC) and deceleration capacity (DC) (34).

### 2.9 Statistical analysis

Statistical analyses were conducted using R software (R Development Core Team, version 4.1.0, New Zealand). The significance level was set at *p* < 0.05. we did not base the choice of statistical method solely on the results of the Shapiro Wilk test. Instead, to apply a suitable for small samples without assuming normality, we conducted all comparisons using the Wilcoxon signed-rank test. Nonparametric data are presented as median and interquartile range (IQR). The IQR was calculated as the difference between the 75th and 25th percentile values.

## 3 Results

In general, it was observed that in the post-administration period, DXR generated changes in blood pressure tests, ECG, echocardiography, Holter ECG, and HRV measurements, which are summarized in Table 1.

**Table 1:** Blood pressure, electrocardiograph, echocardiography, and heart rate variability variables pre and post of doxorubicin administration.

Indices	Units	Pre	Post	*p* value
		Median (range)	Median (range)	
HR	bpm	107.5 (84.5–121.5)	114.8 (98.7–121.5)	0.69
SBP	mmHg	133.2 (119.3–132.5)	131.5 (126.5–132.5)	0.84
QT interval	msec	0.22 (0.19–0.22)	0.19 (0.19–0.20)	0.29
FS	%	34.4 (33.9–42.8)	37.8 (34.7–42.8)	0.73
S′ sep	cm/s	7.5 (6.8–8.0)	6.3 (6.0–8.0)	0.46
S′ lat	cm/s	8.3 (7.3–9.0)	8.4 (7.6–8.6)	1.0
GCS Basal EN	%	−25.1 (−23.8 – −7.0)	−21.3 (−21.9 – −20.3)	0.032*
GCS Basal EP	%	−10.1 (−10.1 – −10)	−9.6 (−9.7 – −9.2)	0.35
GCS Basal MID	%	−15.6 (−15.8 – −15.3)	−14.7 (−14.7 – −14.5)	0.11
GCS Mid EN	%	−25.5 (−27.5 – −25.0)	−26.2 (−29.5 – −25.9)	0.75
GCS Mid EP	%	−10.1 (−10.1 – −8.6)	−9.0 (−9.8 – −8.3)	0.40
GCS Mid MID	%	−16.4 (−17.0 – −16.4)	−16.4 (−17.3 – −15.2)	0.92
GCS Apical EN	%	−24.5 (−25.6 – −24.2)	−26.0 (−26.9 – −23.3)	1.0
GCS Apical EP	%	−8.0 (−8.4 – −7.5)	−7.9 (−7.9 – −7.8)	0.69
GCS Apical MID	%	−14.8 (−15.2 – −14.5)	−14.9 (−15.2 – −13.5)	0.68
GLS EN	%	−19.1 (−21.3 – −17.5)	−18.0 (−19.3 – −17.3)	0.68
nHF	ms^2^	0.25 (0.21–0.27)	0.21 (0.18–0.23)	0.42
LF/HF	ms^2^	0.77 (0.58–0.93)	1.2 (0.79–1.7)	0.31
SDNN	ms	199 (183–207)	206 (127–212)	0.84
RMSSD	ms	183 (178–204)	165 (96.9–171)	0.056
SD1	ms	130 (126–144)	117 (68.6–121)	0.056
SD2	ms	249 (225–254)	263 (166–274)	1.0
SD1/SD2	%	0.58 (0.57–0.60)	0.42 (0.40–0.45)	0.008*
SD2/SD1	%	1.8 (1.76–1.82)	2.5 (2.3–2.7)	0.008*
AC		−11 (−12.7 – −10.7)	−11.9 (−11.9 – −9.6)	0.53
DC		9.8 (8.8–12.2)	12.1 (8.9–13.2)	1.0

HR, heart rate; SBP, systolic blood pressure; FS, fractional shortening; GCS, global circumferential strain; EN, endocardium; EP, epicardium; MID, mid-layer between endocardium and epicardium; GLS, global longitudinal strain; nHF, normalized high frequency; LF, low frequency; HF, high frequency; SDNN, standard deviation on NN intervals; RMSSD, root mean squared of successive RR intervals; SD1, standard deviation of the distance of each point from the y = *x* axis of a Poincaré plot; SD2, standard deviation of the distance of each point from y = x + mean R-R interval of a Poincaré plot; AC, acceleration capacity; DC, deceleration capacity. *p value < 0.05.

### 3.1 Animals

Six female Beagles were included in this study, with ages ranging from 1 to 1.5 years (mean [standard deviation]: 1.2 years [0.24]), and weights ranging from 7 kg to 9.3 kg (mean [standard deviation]: 8.1 kg [0.74]). One dog was excluded from the analysis due to missing data (completely at random [MCAR]) because it died suddenly on day 12 after a cumulative DXR dose of 150 mg/m^2^.

### 3.2 Blood pressure test, electrocardiography, conventional echocardiography

No significant differences were found in systolic blood pressure (SBP) between the Pre and Post conditions (median [interquartile range], 133.2 mmHg [119.3–132.5] vs. 131.5 mmHg [126.5–132.5], *p* = 0.84). ECG showed sinus rhythm in both Pre and Post. Additionally, the QT interval was not significantly different between Pre and Post (0.22 msec [0.19–0.22] vs. 0.19 msec [0.19–0.20], *p* = 0.29). Conventional echocardiography showed no significant difference between Pre and Post for FS (34.4% [33.9–42.8] vs. 37.8% [34.7–42.8], *p* = 0.73), S′ septal (7.5 cm/s [6.8–8.0] vs. 6.3 cm/s [6.0–8.0], *p* = 0.46), and S′ lateral wall (8.3 cm/s [7.3–9.0] vs. 8.4 cm/s [7.6–8.6], *p* = 1.0).

### 3.3 Two-dimensional speckle-tracking echocardiography

In GCS, Basal EN was significantly lower Post than Pre (−25.1% [−23.8 – −7.0] vs. −21.3% [−21.9 – −20.3], *p* = 0.032). No significant difference between Pre and Post for Basal EP (−10.1% [−10.1 – −10] vs. −9.6% [−9.7 – −9.2], *p* = 0.35) and Basal MID (−15.6% [−15.8 – −15.3] vs. −14.7% [−14.7 – −14.5], *p* = 0.11) were observed. Mid EN, Mid EP, and Mid MID were not significant between Pre and Post (Mid EN: −25.5% [−27.5 – −25.0] vs. −26.2% [−29.5 – −25.9], *p* = 0.75) (Mid EP: −10.1% [−10.1 – −8.6] vs. −9.0% [−9.8 – −8.3], *p* = 0.40) (Mid MID: −16.4% [−17.0 – −16.4] vs. −16.4% [−17.3 – −15.2], *p* = 0.92). Furthermore, Apical EN, Apical EP, and Apical MID did not show any significant differences (Apical EN: −24.5% [−25.6 – −24.2] vs. −26.0% [−26.9 – −23.3], *p* = 1.0) (Apical EP: −8.0% [−8.4 – −7.5] vs. −7.9% [−7.9 – −7.8], *p* = 0.69) (Apical MID: −14.8% [−15.2 – −14.5] vs. −14.9% [−15.2 – −13.5], *p* = 0.68). In GLS, no significant difference was found between Pre and Post for EN (−19.1% [−21.3 – −17.5] vs. −18.0% [−19.3 – −17.3], *p* = 0.68).

### 3.4 Linear heart rate variability

There were no significant differences in the linear HRV indices between the Pre and Post conditions. Frequency analysis nHF was not significantly different between Pre and Post (0.25 ms^2^ [0.21–0.27] vs. 0.21 ms^2^ [0.18–0.23], *p* = 0.42). Meanwhile, no significant difference was found in LF/HF between Pre and Post (0.77 ms^2^ [0.58–0.93] vs. 1.2 ms^2^ [0.79–1.7], *p* = 0.31). Time domain analysis showed no significant difference between Pre and Post for both SDNN (199 ms [183–207] vs. 206 ms [127–212], *p* = 0.84) and RMSSD (183 ms [178–204] vs. 165 ms [96.9–171], *p* = 0.056).

### 3.5 Nonlinear heart rate variability

SD1/SD2 was significantly lower in the Post group than in the Pre group, whereas SD2/SD1 was significantly higher in the Post group. Therefore, sympathetic activity was observed (Figure 1). In the geometric analysis, both SD1 and SD2 showed no significant difference (SD1: 130 ms [126–144] vs. 117 ms [68.6–121], *p* = 0.056) (SD2: 249 ms [225–254] vs. 263 ms [166–274], *p* = 1.0). However, SD1/SD2 was significantly lower in Post than Pre (0.58% [0.57–0.60] vs. 0.42% [0.40–0.45], *p* = 0.008). SD2/SD1 was also significantly higher in Post than Pre (1.8% [1.76–1.82] vs. 2.5% [2.3–2.7], *p* = 0.008). Additionally, AC and DC in PRSA showed no significant difference between Pre and Post (AC: −11 [−12.7 – −10.7] vs. −11.9 [−11.9 – −9.6], *p* = 0.53) (DC: 9.8 [8.8–12.2] vs. 12.1 [8.9–13.2], *p* = 1.0).

## 4 Discussion

### 4.1 Possible explanation and implications

FS was consistent with predictions and showed no change (22). FS is known to be influenced by loading conditions (6). In this study, the lack of a significant decrease in systolic blood pressure due to DXR cardiotoxicity is thought to be due to an increase in blood volume, peripheral vascular resistance, blood viscosity, and others factors, which may have increased systolic blood pressure. Additionally, FS, which is widely used in veterinary medicine, and ejection fraction (EF), which is widely used in human medicine, differ in their calculation methods. FS is calculated using left ventricular diameter, while EF uses left ventricular volume. This difference in calculation methods may also have influenced the results of this study. The Simpson method is one of the methods used to calculate EF and is considered an ideal method that provides highly accurate measurements with minimal measurement error due to the shape of the left ventricle (35). Although it is possible to use EF in veterinary medicine, it is difficult to display the left apical view, so there is a high probability that the measurement becomes more complicated (36). Considering the influence of various biases and the lack of a significant difference in the results of this study, it is believed that the use of FS for early detection of cardiovascular disease would be difficult. Linear analysis of HRV in dogs may be susceptible to several biases, including those related to respiration, duration, and respiratory arrhythmia (27, 37). In addition, life is inherently nonlinear and possesses properties that cannot be analyzed by linear analysis. Therefore, it is appropriate to use nonlinear analysis for HRV as well. Thus, there was no significant change in linear HRV, whereas the Poincaré plot, a nonlinear analysis, showed increased sympathetic activity in the Post group, as expected. On the other hand, non-linear HRV showed no significant difference between AC and DC, which was contrary to expectations. Dogs with respiratory arrhythmias may not have been appropriate indicators for assessing the cardiotoxicity of DXR due to the acceleration and deceleration of the heartbeat associated with breathing.

Basal EN of 2D-STE showed decreased systolic function. This is thought to be due to the increased sympathetic nerve activity induced by DXR administration, which increased the wall stress derived from Laplace’s law in Basal region, which has a larger radius and thinner wall thickness than Mid and Apical regions, thus placing more stress on the myocardium. The changes in the EN were also thought to be due to the fact that it is farthest from the coronary artery. EN of GLS is believed to be the first of the 2D-STEs to show changes in systolic function (6). This is because longitudinal strain has been suggested to be worse than circumferential strain in the early phase of ischemia due to the longitudinal orientation of endocardial fibers (38). In the present study, EN of GLS showed no change in systolic function of the Post group. This may reflect the fact that although myocardial strain is increased, oxygen demand to the myocardium is maintained, and thus ischemia may not have been occurred. However, 2D-STE may have influenced changes in Basal EN values because it is a method prone to large measurement errors (6).

Therefore, the Poincaré plot, the nonlinear HRV method used in this study, is a favorable test for early detection of heart disease in dogs, considering its alignment with the essence of life and its low susceptibility to hemodynamic and measurement errors.

### 4.2 Limitation

A limitation of this study is the lack of consideration of breed differences. For example, it has been suggested that brachycephalic breeds may exhibit higher cardiac vagal activity compared to non-brachycephalic breeds (39). In addition, the heterogeneity of the rearing environment of the experimental animals is another limitation. If future studies are conducted with domestic dogs living in human households, HRV may be influenced by the human life rhythm, potentially impacting HRV measurements. In humans, cardiotoxicity rarely manifests acutely but rather becomes a problem several years after chemotherapy, as seen in cancer survivors (40). There are no reports on the long-term evaluation of heart rate variability after chemotherapy in veterinary medicine, indicating the need for further research in this area. Therefore, the lack of a long-term evaluation is also considered a limitation of this study.

## 5 Conclusion

In conclusion, SD1/SD2 and SD2/SD1 significantly reflected the increased sympathetic activity induced by DXR cardiotoxicity. It is worth noting the nonlinear HRV Poincaré plot as a test for early detection of DXR-induced myocardial damage. This finding suggests that the use of Poincaré plots for nonlinear HRV assessment is an important step toward promoting early detection, early treatment intervention for various heart diseases and improving clinical outcomes. However, due to the l sample size, the present results should be regarded as exploratory, and further validation in larger populations is required.

## Data Availability

The original contributions presented in the study are included in the article/supplementary material, further inquiries can be directed to the corresponding author.
